# Optimizing regional innovation ecosystems through actor-environment coevolution: A dynamic configurational analysis from a CAS perspective

**DOI:** 10.1371/journal.pone.0341011

**Published:** 2026-01-21

**Authors:** Hanjun Pang, Yiling Jiang, Lei Wu, Lu Wang

**Affiliations:** 1 Institute for Chengdu-Chongqing Economic Zone Development, Chongqing Technology and Business University, Chongqing, China; 2 Chongqing University of Education, Chongqing, China; 3 School of Business Administration, Chongqing Technology and Business University, Chongqing, China; National Center for Chronic and Noncommunicable Disease Control and Prevention, Chinese Center for Disease Control and Prevention, CHINA

## Abstract

The interaction among innovation actors and environmental uncertainties has intensified the complexity of the evolution and drivers of regional innovation ecosystems, with profound implications for regional economies. This research aims to investigate the evolution and impact mechanisms of regional innovation ecosystems through the complex adaptive systems perspective. Employing an integrated methodology, we combine quantitative performance evaluation with dynamic fuzzy-set qualitative comparative analysis to examine 30 Chinese provinces. Specifically, we investigate how innovation actors (governments, enterprises, universities) and key evolving institutional environment (openness, human capital and innovation platform) interact, adapt, and collaborate to shape innovation performance. We also analyze temporal changes and regional disparities. Our findings reveal significant differences in regional innovation performance across China’s eastern, central, and western regions, though all regions exhibit yearly improvement. Strong government investment in innovation is a necessary condition for high-level regional innovation, while weak enterprise R&D inputs are a necessary condition for low-level regional innovation. The analysis identifies five distinct configurations driving high innovation performance and four configurations associated with low-level regional innovation. The between-group consistency reveals that regional innovation increasingly depends on complex interactions among multiple actors and environments. The within-group consistency indicates that in developed eastern regions, innovation is driven by multiple innovation actors and environments, with high external knowledge dependence due to openness. In contrast, innovation in underdeveloped western regions mainly relies on internal factors like government and enterprise innovation investments. Case studies include both developed and lagging regional innovation ecosystems. Our research offers a novel theoretical perspective for understanding regional innovation ecosystem evolution. The findings provide policymakers with a scalable framework to tailor region-specific innovation strategies across diverse contexts, with insights applicable to innovation research in other regions.

## 1. Introduction

Innovation is an imperative for productivity, long-term growth, and well-being in modern economies on a global scale [1]. In recent years, the innovation environment has mixed, with significant opportunities and sizeable challenges on the horizon. This has led to increased innovation uncertainty and global innovation polarization [2]. As regional science and innovation systems continue to evolve, and the practical promotion of interactive innovation through geographical proximity gains traction, regional innovation policies have garnered increased attention. However, the formulation and implementation of regional innovation policies remain challenging due to the complex collaboration among innovation actors and environment [3]. Regional innovation is not only a core driving force for regional economic development, but also important to a nation’s overall competitiveness [4,5]. Considering the significant disparities among different regions, blanket innovation policies of the nation frequently fail to be effective. Understanding the diversity and dynamic within a region is essential for developing an effective regional policy framework [6]. Therefore, the critical factor in promoting regional innovation performance lies in how key innovation actors adapt to and respond to these changing contexts.

As the world’s second-largest economy, China possesses diverse regional resource endowments and local conditions [7]. In recent years, China has actively pursued an innovation-driven development strategy [8], significantly increasing research and development (R&D) investments and achieving major technological breakthroughs in emerging industries like renewable energy and artificial intelligence. However, as China’s innovation capabilities grow, regional disparities persist, making coordinated regional development a critical policy priority [9]. Meanwhile, other global regions are also shifting toward context-specific approaches to enhance the effectiveness of regional innovation ecosystems, moving away from uniform “one-size-fits-all” policies.

Extant research has confirmed the systematic and dynamic nature of regional innovation processes. For example, regional innovation system theory highlights innovation actors’ collaborative importance [10]. The regional innovation ecosystem uses the innovation process as an analogy to an ecosystem, emphasizing the dynamic adaptability and coordination of innovation actors with their environment [11]. Research has examined regional innovation characteristics and explored its determinants comprehensively [12,13]. However, a deeper understanding of how key actors adapt to and co-evolve with their regional innovation environments is needed, particularly considering the effects of path dependence in different regional contexts [14]. These dimensions are critical for explaining persistent regional innovation disparities and designing effective innovation policies.

To address these issues, according to the complex adaptive systems (CAS) theory, we employ an integrated approach combining quantitative assessment and dynamic fuzzy-set qualitative comparative analysis (fsQCA) to examine the evolution and impact mechanisms of regional innovation ecosystems. Specifically, we investigate how innovation actors (governments, enterprises, universities) and key evolving institutional environment (openness, human capital and innovation platform) interact, adapt, and collaborate to shape innovation performance. Moreover, we examine temporal changes and regional differences. This study aims to answer the following questions: How is China’s regional innovation influenced by the co-evolution of innovation actors and environments? What are the temporal variations and regional differences in this influence? We analyze both developed and lagging regional innovation ecosystems, and divide provinces into eastern, central, and western regions.

This study makes several contributions. First, by drawing on CAS theory, we highlight the dynamic interactions, adaptations, and synergies between innovation actors and institutional environments. This provides a novel theoretical perspective for understanding the evolution of regional innovation ecosystems. Second, to reveal systemic complexity, we integrate quantitative evaluation with fsQCA. This methodology identifies multiple concurrent causes, revealing the temporal evolution of factor configurations to addressing limitations in existing research on evolutionary processes. Third, our research incorporates temporal and spatial dimensions into the analytical framework. This not only answers the question of what, but also explores how changes occur and why differences exist within local contexts.

The remainder of this paper is organized as follows: Section 2 reviews the related literature. Section 3 outlines the materials and methods. Section 4 analyzes the results. Section 5 discusses the findings. Section 6 presents the conclusion, implications, and generalizations of the research.

## 2. Literature review

### 2.1 Global innovation paradigms and theoretical evolution

The evolution of innovation theory toward systemic models was spurred by a growing recognition of the complexity and uncertainty that linear models failed to capture. Furthermore, Freeman [15] introduced the concept of innovation networks, emphasizing the critical role of social relationships and knowledge flows among diverse actors in driving innovation. The triple helix model emphasizes the overlapping roles and connections among universities, industry, and government, which jointly promote innovation through spiral interactions [16,17]. Over the past decade, scholars have noted that the spatial configuration of innovation systems has become increasingly complex, with networks of actors and institutional contexts originating from various regions and spanning multiple spatial scales [18–20]. Diverse analytical approaches have been initiated to conceptualize the increasingly significant international linkages within innovation systems [19,21]. Therefore, some scholars conceptualized the global innovation system and proposed a framework for technological innovation in the transnational context [21]. The global innovation subsystem is no longer based on preset geographical boundaries but on a network of actors and institutions that participate in creating specific system resources, such as knowledge, market access, capital investment, and technological legitimacy [21–23].

Global innovation research has affirmed the significance of the systems perspective. Nevertheless, it frequently remains overly macroscopic and lacks the explanatory capacity for the specific, interaction mechanisms and dynamic evolution within the system.

### 2.2 National innovation system

The National Innovation System (NIS) concept emphasizes the systematic and multi-actor interaction of innovation processes at the national level. Freeman provides a classic definition in his work: “the network of institutions in the public and private sectors whose activities and interactions initiate, import, modify and diffuse new technologies” [24]. It explicitly addresses what has been neglected in early models of technological change: the conscious “intangible” investment in technological learning activities involving various institutions (primarily business companies, universities, other educational and training institutions, and governments), their connections, and the associated incentive structures and capabilities [25]. This theoretical framework effectively accounts for the enduring disparities in innovation patterns and performance across different countries [26].

However, regarding the adequacy of the NSI analysis, one issue is the appropriateness of using the national level as the starting point for analyzing innovation processes. This is particularly pertinent given the necessity to disentangle complex interrelationships, as the selected models for testing are no longer linear but interactive [4].

### 2.3 Regional innovation system: actors and milieu

Cooke’s [4,10] regional innovation system framework emphasizes the significance of interactions among innovation actors, and the roles of financial support, institutionalized learning, and production culture in regional innovation. It suggests strengthening regional capacity building to promote systemic learning and interactive innovation [4]. Regional innovation actors mainly include government, enterprises and universities [4]. Governments, acting as regulatory and resource allocating actors, coordinate regional innovation by formulating policies and institutional protections to channel resources into strategic sectors [27]. The agglomeration of enterprises and industries enhances innovation via knowledge spillovers facilitated by geographical proximity and collaborative economies of scale [28]. Universities, as the primary generators of knowledge, produce geographically restricted spillovers through public interest research [29]. Moreover, the transfer of knowledge from universities to private enterprises is of great significance within universities as it is part of their third mission [30,31].

The innovative milieu framework emphasizes regional innovation’s sociocultural embeddedness, stating that factors such as institutional thickness, cultural norms, and social capital mediate interactions among innovation actors [32]. This view revolutionized regional development paradigms by showing how localized innovation climates form distinct technological advancement patterns [33]. Importantly, the milieu approach analyzes innovation variances from two perspectives: macro-level cultural archetypes that shape knowledge appropriation [34,35] and micro-level social capital mechanisms that govern collaborative learning [36,37]. These forces influence innovation performance by shaping the interactions among firms, policymakers, and research institutions [33].

Although regional-level research has explored specific factors, it lacks dynamic longitudinal comparisons across regions. Most analytical methods assume linear causal relationships, making it difficult to capture the complex, nonlinear interactions between these factors. Furthermore, there is insufficient empirical evidence to examine how actors and environments co-evolve.

### 2.4 The theoretical perspective: complex adaptive systems

CAS theory, based on interdisciplinary studies of biology, computer science, and economics, offers a framework for understanding how interconnected actors interact and self-organize to generate emergent system-level behaviors [38]. Different from reductionist paradigms, CAS methodology combines qualitative reasoning with quantitative modeling, balancing micro-level emergent patterns and macro-level agent behaviors and synthesizing reductionist and holistic perspectives [38,39]. CAS are characterized by four core principles: (1) adaptation, where agents evolve strategies in response to environmental feedback; (2) non linearity, as small perturbations may trigger disproportionate outcomes; (3) emergence, whereby macro-level patterns arise unpredictably from micro-level interactions; and (4) path dependence, where historical contingencies constrain future trajectories [40,41].

Due to its distinctive characteristics, the CAS theory possesses functional advantages, demonstrating tremendous potential for analyzing complex systems across various domains such as the economy, ecology, society, management, and military affairs. By adopting CAS principles, scholars explore dynamic strategies for navigating turbulent environments. In regional science and innovation studies, the theory explains how regions evolve as adaptive networks of firms, institutions, and policies, with innovation pathways emerging from multi-actor collaborations and resource reconfiguration [42]. However, the application of this theory in the field of regional innovation remains limited. In rapidly changing environments, where regional innovation activities are becoming increasingly systematic and complex, examining the development of regional innovation ecosystems from the CAS perspective aligns more closely with reality and holds significant theoretical value.

### 2.5 Research gap and objectives

In summary, while existing literature on regional innovation is abundant and detailed, several limitations remain. First, existing empirical studies have insufficiently examined the dynamic co-evolution between innovation actors and their environment in influencing regional innovation. Although the dynamism of regional innovation has been thoroughly revealed and analyzed, there remains room for exploration regarding its underlying mechanisms. Second, most empirical studies focus on single factor net effects, neglecting the complex, non-linear interactive relationships among multiple actors, which contradicts real world regional innovation ecosystem characteristics. Furthermore, policy recommendations overlook regional contextual differences and innovation path dependencies, so they are overly generalized and fail to fit specific regional ecosystem development stages and internal conditions.

Therefore, according to the CAS theory, we assessed the regional innovation performance of 30 provinces and cities in China and analyzed their spatial-temporal heterogeneity. We utilized dynamic QCA to investigate how configurations of innovation actors and environmental conditions produce nonlinear effects on regional innovation ecosystems. This method surpasses the static constraints of traditional QCA by integrating temporal analysis. The configuration paths of regional innovation in developed and underdeveloped regions are examined based on their distinct contexts.

## 3. Materials and methods

### 3.1 Methods

#### 3.1.1 Evaluation method.

In this study, to account for disparities in the magnitude of each indicator, the data were standardized using the min-max method to enable fair comparisons across regions. Entropy-based weights were assigned to each secondary indicator, and the overall regional innovation performance score was calculated.

The entropy method is an objective approach used for assigning weights in multi-criteria evaluations, particularly when there are significant disparities between indicators. This method calculates the information entropy for each indicator to determine its weight. The entropy value reflects the degree of uncertainty: higher entropy indicates that the indicator provides less useful information, thus contributing less to the overall evaluation. Conversely, lower entropy indicates more informative data, making the indicator more influential in the assessment.

To eliminate scale differences among indicators, data is standardized. The Min-Max normalization method is used to transform the values to a 0–1 range:


$$X_{ij}^{\prime }=\frac{X_{ij}-X_{i,\min}}{X_{i,\max}-X_{i,\min}}$$ (1)

Where $X_{ij}$ represents the original value of the i -th region for the j -th indicator, and $X_{i,\min}$ and $X_{i,\max}$ are the minimum and maximum values for the i -th region and j -th indicator. After standardization, the proportion for each indicator is calculated as follows:


$$p_{ij}=\frac{{X^{\prime }}_{ij}}{\sum {\mathrm{\backslash nolimits}}_{i=1}^{n}{X^{\prime }}_{ij}}$$ (2)

Where $p_{ij}$ represents the proportion of the i -th region for the j -th indicator, and n is the number of regions. The entropy value for each indicator is computed using the formula:


$$e_{j}=-\frac{\text{1}}{\ln(n)}\sum_{i=1}^{n}p_{ij}\ln(p_{ij})$$ (3)

Where $e_{j}$ is the entropy value for the j -th indicator, and n is the number of regions. A higher entropy indicates less variation in the data, resulting in a lower weight for that indicator. The weight for each indicator is derived from its entropy value using the following formula:


$$w_{j}=\frac{1-e_{j}}{\sum {\mathrm{\backslash nolimits}}_{j=1}^{m}\left(1-e_{j}\right)}$$ (4)

Where $w_{j}$ is the weight of the j -th indicator, and m is the total number of indicators. Finally, the overall innovation performance for each region is calculated by summing the weighted standardized values of each indicator:


$$S_{i}=\sum_{j=1}^{m}w_{j}X_{ij}^{\prime }$$ (5)

Where $S_{i}$ is the regional innovation performance for the i -th region, and $X_{ij}^{\prime }$ is the standardized value for the i -th region on the j -th indicator.

#### 3.1.2 Dynamic fsQCA method.

This study employs dynamic fsQCA [43], an extension of traditional fsQCA that incorporates a temporal dimension. Unlike conventional fsQCA, which analyzes cross-sectional data and lacks time sensitivity [44], dynamic fsQCA evaluates pooled consistency, between-group consistency, and within-group consistency. It utilizes Between-group Consistency Adjusted Distance (BECONS Adj-distance) and Within-group Consistency Adjusted Distance (WICONS Adj-distance) to track changes in consistency over time and across cases [43,45]. The fsQCA combines Boolean algebra, set theory, and fuzzy-set logic to identify necessary/sufficient condition configurations for an outcome [46]. It supports inductive analysis [47], asymmetric causality, and equifinality [48].

We adopt dynamic fsQCA for three reasons: First, regional innovation involves nonlinear, multi-factor interactions, which fsQCA captures through configuration analysis. Second, innovation drivers evolve over time; panel data analysis reveals trends. Third, geographic differences necessitate spatial-temporal analysis.

### 3.2 Data and sample selection

This study uses data from 30 provinces and cities in China, excluding Hong Kong, Macao, Taiwan, and Tibet due to missing data. The data cover the period from 2016 to 2022, with annual data available for each province and city. 2016 was chosen as the starting point as it marked the start of China’s 13th Five-Year Plan for economic and social development. This plan elevated the “innovation-driven development strategy” to a new level and provided a policy background for observing the systematic evolution of regional innovation ecosystems. Moreover, the 7 year time span provides sufficient data support for analyzing spatial-temporal changes with the dynamic fsQCA method.

Due to China’s vast geography, significant regional disparities exist in innovation performance, driven by varying political, economic, cultural, and social factors. The 30 provinces and cities include diverse regions, such as the economically advanced coastal areas, the less-developed inland western regions, and areas with distinct social and cultural characteristics. These provinces and cities are grouped into three regions based on their geographic location and economic development. Eastern Region: Beijing, Tianjin, Hebei, Liaoning, Shanghai, Jiangsu, Zhejiang, Fujian, Shandong, Guangdong, and Hainan; Central Region: Shanxi, Inner Mongolia, Jilin, Heilongjiang, Anhui, Jiangxi, Henan, Hubei, and Hunan; Western Region: Guangxi, Chongqing, Sichuan, Guizhou, Yunnan, Shaanxi, Gansu, Qinghai, Ningxia, and Xinjiang.

The data were sourced from the China Statistical Yearbook, China Science and Technology Statistical Yearbook, China Torch Statistical Yearbook, and statistical yearbooks from the provinces and cities. Considering that R&D investment, patent applications to authorization, and other factors have a time-lagged effect on regional innovation performance [48], the outcome variable was treated with a two-year lag relative to the conditional variable. Specifically, the conditional variable was evaluated from 2014 to 2020 and the outcome variable was evaluated from 2016 to 2022.

### 3.3 Variables

#### 3.3.1 Evaluation index of regional innovation performance.

Regional innovation is a multidimensional and dynamic process. Previous studies have commonly used patent counts to measure regional innovation performance. While patents are a well-established indicator of technological strength [50], they have limitations. Patent counts do not fully capture the complexity of innovation, and regional innovation is difficult to measure using a single quantity [51,52]. In addition to the technological innovation represented by patents, innovation activities also include product innovation [53,54] and academic innovation [55]. According to the research of Zhang et al [56], we propose evaluating regional innovation performance based on three dimensions: academic innovation, technological innovation, and product innovation. We measured academic innovation by the number of scientific papers published, technological innovation by the number of patents granted, and product innovation by revenue from new product sales. The evaluation indicator index for regional innovation performance is shown in Table 1.

**Table 1:** The comprehensive evaluation of regional innovation performance.

Primary Indicators	Secondary Indicators	Data Description
	Technological Innovation	Number of patents granted
Regional Innovation Performance	Product Innovation	Revenue from the sale of new products by industrial enterprises
	Academic Innovation	Number of scientific papers published

#### 3.3.2 Conditions.

The selection of conditional variables in this study considered two aspects. First, CAS theory emphasizes the important influence of innovation actors and their interaction and cooperation on regional innovation performance. Referring to the conceptual framework of government–industry–university provided by the “triple helix” theory [17], the innovation actors considered in this study include the government, enterprises, and universities. Second, the innovative milieu approach proposes that some non-technological milieu contexts of a region can explain differences in the level of innovation among regions. Accordingly, in combination with China’s regional innovation practices, we propose opening, human capital, and innovation services as key innovative milieu factors.

As the innovation actor, some studies have used local government science and technology expenditure to reflect the government’s financial support and emphasis on local innovation activities [7,57]. In general, the flow and investment of R&D resources, financial and human are considered important direct inputs for the innovation process [58,59]. Zhang [56] and Huang [48] used enterprise R&D internal expenditure and enterprise R&D personnel full-time equivalent expenditure as enterprise innovation variables. Similarly, we use university R&D internal expenditure and university R&D personnel full-time equivalent expenditure as university innovation variables.

**Fig 1:**
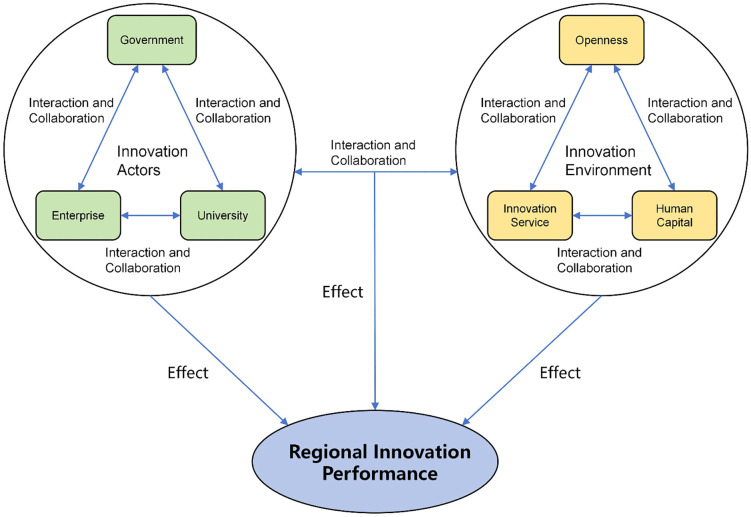
Conditions framework.

Regarding the regional innovation milieu, research has shown that it is more important for a region’s economic development to gain a first-mover advantage by opening up to the outside world than to develop a closed, defensive knowledge and technology protection system [60]. Regional foreign trade dependence is considered a representative indicator of open milieu, often measured as the proportion of goods imports and exports to GDP [56]. Although foreign direct investment can bring new knowledge and technology to a region, it also represents the degree of regional openness [54,61]. Therefore, the open milieu is measured using two indicators: the proportion of goods imports and exports of GDP and the proportion of foreign direct investment of GDP. Business incubators are important innovation service platforms for enterprise innovative development [62,63]. This factor is measured using the number of science and technology business incubators and the amount of investment and financing received by science and technology business incubators. Higher education is an important prerequisite for technologically lagging regions to catch-up, and is closely related to the importance of interactive learning as a basis for innovation and change in modern developed economies. Some studies have shown that if sufficient human capital is accumulated, it can promote local innovative activities and economic growth [64,65]. Regarding the level of human capital, this was operationalized as the average years of schooling, with reference to related studies [66,67]. For multiple secondary indicators, the entropy method was applied to assign weights and calculate the total indicator value. The conceptual framework of variables in this study is illustrated in Fig 1.

## 4. Results

### 4.1 Regional innovation performance evaluation results

As shown in Table 2, the rankings of regional innovation performance from 2016 to 2022 reveal that the top five performing provinces and cities are Guangdong, Jiangsu, Zhejiang, Shandong, and Beijing, which are all located in the economically advanced eastern coastal region. In contrast, the lowest ranking provinces and cities include Gansu, Xinjiang, Ningxia, Hainan, and Qinghai. Except for Hainan, most of these are located in the less developed western region. Regional innovation performance improved across all provinces and cities over time, with 2022 values surpassing those of 2016. This indicates that China’s regional innovation levels have steadily increased, reflecting the success of the government’s innovation and coordinated development strategies.

**Table 2:** The regional innovation performance by provinces (2016–2022).

	2016	2017	2018	2019	2020	2021	2022	Mean	Rank
Guangdong	0.4429	0.5276	0.6495	0.7042	0.8170	0.9453	0.9182	0.7150	1
Jiangsu	0.4541	0.4649	0.5119	0.5392	0.7069	0.8059	0.8529	0.6194	2
Zhejiang	0.3259	0.3245	0.3772	0.4025	0.4737	0.5846	0.6143	0.4433	3
Shandong	0.2317	0.2566	0.2578	0.2554	0.3332	0.4669	0.5609	0.3375	4
Beijing	0.2053	0.2189	0.2289	0.2432	0.2539	0.3001	0.2903	0.2486	5
Shanghai	0.1855	0.2032	0.2153	0.2317	0.2529	0.2957	0.3034	0.2411	6
Hubei	0.1527	0.1626	0.1856	0.1961	0.2143	0.2742	0.2954	0.2116	7
Anhui	0.1247	0.1365	0.1523	0.1577	0.1978	0.2439	0.2652	0.1826	8
Hunan	0.1318	0.1435	0.1448	0.1542	0.1697	0.2073	0.2334	0.1692	9
Sichuan	0.1227	0.1297	0.1489	0.1518	0.1732	0.2082	0.2185	0.1647	10
Henan	0.1222	0.1297	0.1514	0.1461	0.1748	0.2038	0.2041	0.1617	11
Fujian	0.0884	0.0954	0.1179	0.1217	0.1465	0.1664	0.1630	0.1285	12
Hebei	0.0822	0.0912	0.1002	0.1140	0.1354	0.1677	0.1650	0.1223	13
Shaanxi	0.0947	0.0959	0.1106	0.1217	0.1283	0.1508	0.1498	0.1217	14
Liaoning	0.0972	0.0981	0.1099	0.1098	0.1233	0.1369	0.1358	0.1159	15
Jiangxi	0.0626	0.0695	0.0849	0.1029	0.1223	0.1507	0.1583	0.1073	16
Chongqing	0.0918	0.0912	0.0886	0.0935	0.1101	0.1318	0.1308	0.1054	17
Tianjin	0.0929	0.0800	0.0915	0.0933	0.1006	0.1231	0.1141	0.0994	18
Jilin	0.0630	0.0609	0.0520	0.0637	0.0660	0.0739	0.0657	0.0636	19
Heilongjiang	0.0483	0.0521	0.0550	0.0610	0.0639	0.0694	0.0679	0.0596	20
Guangxi	0.0451	0.0465	0.0463	0.0490	0.0635	0.0740	0.0720	0.0566	21
Shanxi	0.0300	0.0356	0.0431	0.0456	0.0563	0.0659	0.0686	0.0493	22
Yunnan	0.0345	0.0324	0.0386	0.0411	0.0443	0.0498	0.0502	0.0416	23
Guizhou	0.0244	0.0258	0.0298	0.0362	0.0432	0.0467	0.0443	0.0358	24
Neimenggu	0.0232	0.0247	0.0241	0.0249	0.0297	0.0351	0.0470	0.0298	25
Gansu	0.0227	0.0237	0.0244	0.0271	0.0300	0.0366	0.0376	0.0289	26
Xinjiang	0.0191	0.0181	0.0185	0.0176	0.0202	0.0275	0.0299	0.0216	27
Ningxia	0.0052	0.0082	0.0086	0.0091	0.0103	0.0115	0.0171	0.0100	28
Hainan	0.0037	0.0043	0.0046	0.0053	0.0076	0.0118	0.0125	0.0071	29
Qinghai	0.0002	0.0006	0.0014	0.0022	0.0045	0.0050	0.0063	0.0029	30

**Fig 2:**
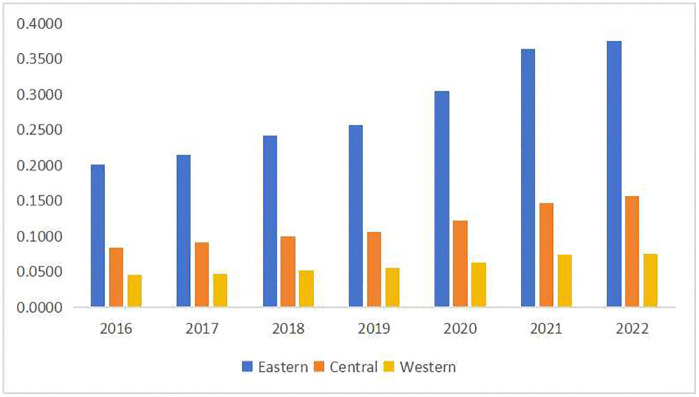
Regional innovation performance over time in three regions of China.

As illustrated in Fig 2, when comparing innovation performance across regions, the eastern region consistently outperforms the central and western regions. The gap is particularly pronounced in the eastern region, which has outpaced the central and western regions each year. Despite growth in all regions, the eastern region has seen faster progress, while the western region has lagged behind. This trend aligns with the varying levels of economic development across regions, emphasizing the central role of innovation in driving economic growth. It also suggests that a strong economic foundation and innovation milieu further stimulate regional innovation, creating a virtuous cycle.

### 4.2 Dynamic QCA results

#### 4.2.1 Initial calibration.

Calibration is an important preparatory task in fsQCA. Calibrating the conditional and outcome variables into 0–1 fuzzy-set data allows the variable data to meet analysis criteria [49]. In this study, we use the direct calibration method, in which three qualitative anchors are created for each conditional and outcome variable and then used in a log-odds transformation to calculate the corresponding membership values [49]. These three anchor points represent the degree of full membership, the crossover point, and full non-membership. It is important to define the anchor points for calibration based on the purpose of the study and the data distribution.

According to the variable data characteristics and findings of previous studies [68], the anchor points were set as full membership (fuzzy score = 0.95), crossover (fuzzy score = 0.5), and full non-membership (fuzzy score = 0.05). We primarily used R software for calibration and subsequent data analysis. Three qualitative anchors of each variable are shown in Table 3.

**Table 3:** Three qualitative anchors of each variable.

Variables	Full non-membership	Crossover point	Full membership
Regional Innovation Performance	0.551	0.102	0.006
Government	0.384	0.056	0.003
Enterprise	0.621	0.085	0.002
University	0.541	0.134	0.005
Openness	0.202	0.034	0.008
Innovation Service	0.448	0.041	0.003
Human Capital	0.698	0.321	0.101

#### 4.2.2 Individual necessary conditions.

Necessary condition analysis is an independent procedure of fsQCA in which each conditional variable is analyzed to find the necessary conditions that lead to the occurrence of the outcome. For a condition to be necessary, the level of consistency is generally required to be greater than 0.9 [49] and the affiliation score of the outcome variable must always be lower than the affiliation membership score of the conditional variable under consideration [46]. When the adjusted distance was larger than a certain value, the fluctuation of the consistency level over time across areas needed to be analyzed further [43]. In this study, the consistency-adjusted-distance threshold was set to 0.2. Considering the causal asymmetry of fsQCA [49], two separate results (high and low regional innovation) are presented.

Table 4 shows that for high-level innovation, both strong government and enterprise actors had consistency >0.9 and coverage >0.5. However, only strong government passed the scatterplot test (with most points below the diagonal), making it the necessary condition. For low-level innovation, weak enterprise actors and weak innovation services showed consistency >0.9, but only weak enterprises passed the scatterplot test (with half of points below diagonal), establishing it as the necessary condition for low performance.

**Table 4:** Analysis of necessary conditions.

Conditions	High-Regional Innovation Performance				Low-Regional Innovation Performance			
	Pooled Consistency	Pooled Coverage	BECONS Adj-distance	WICONS Adj-distance	Pooled Consistency	Pooled Coverage	BECONS Adj-distance	WICONS Adj-distance
Government	0.937	0.874	0.032	0.109	0.468	0.564	0.096	0.604
~Government	0.532	0.437	0.192	0.495	0.895	0.948	0.026	0.219
Enterprise	0.911	0.957	0.032	0.161	0.426	0.577	0.019	0.702
~Enterprise	0.597	0.446	0.103	0.414	0.968	0.933	0.022	0.115
University	0.888	0.870	0.061	0.155	0.459	0.580	0.119	0.633
~University	0.572	0.450	0.215	0.437	0.898	0.912	0.042	0.242
Openness	0.800	0.777	0.029	0.282	0.504	0.632	0.103	0.569
~Openness	0.621	0.493	0.055	0.431	0.822	0.841	0.032	0.311
Innovation Service	0.866	0.895	0.138	0.144	0.443	0.592	0.231	0.615
~Innovation Service	0.605	0.457	0.199	0.414	0.921	0.898	0.048	0.161
Human Capital	0.746	0.693	0.064	0.293	0.573	0.687	0.154	0.477
~Human Capital	0.663	0.546	0.115	0.397	0.744	0.791	0.064	0.414

Note: ~ denotes the absence of the condition.

#### 4.2.3 Analysis of sufficiency.

We now analyze the role of conditional variables in generating high and low levels of regional innovation [49], to identify the combinations of variables that satisfy sufficient conditions and determine their effects on the results. For the sufficiency analysis of panel data, the resulting membership scores must always be higher than the membership scores of various combinations of conditions [46].

The first step is to construct a truth table based on Boolean algebraic logic. The truth table lists all logically possible combinations of conditions that lead to high or low levels of regional innovation. During the construction of this truth table, thresholds for consistency, PRI, and n are required. Although some researchers consider a consistency level greater than 0.75 to be acceptable [49], other researchers have proposed a more demanding value of 0.8 to represent an acceptable level [69]. Referring to previous practice and considering the context of this study, we set the consistency threshold to 0.95, PRI to 0.8, and n to 2, which ultimately covered 195 cases. The thresholds were set to be the same for high and low levels of regional innovation.

After constructing the truth table, during the counterfactual analysis, based on the results of the necessary condition analysis, some variable directions are predetermined in the sufficiency analysis process. For high-level regional innovation, the conditional variable representing the government’s innovation actors is set to “1” to indicate the existence of such a necessary condition and other variables are set to “-”, indicating they are not a necessary condition. For low-level regional innovation, the conditional variable representing enterprise innovation actors is set to “0”, to indicate a necessary condition and other variables are set to “-”, indicating they are not necessary. In this study, R software was used to analyze the sufficiency of the conditional configuration, and obtain the corresponding complex, intermediate, and parsimonious solutions. The intermediate solution was used as the main solution and the parsimonious solution was used as the secondary solution to determine the configurations that met the necessary conditions. Based on causal asymmetry, configuration analyzes were conducted separately for high and low level regional innovations. Referring to Fiss [69], a circle representation is used, where • and ⊗ denote the presence and absence of a condition, respectively, and large and small circles denote the core and edge conditions of complex and parsimonious solutions, respectively. The results of the sufficiency condition configurations are presented in Table 5.

**Table 5:** Configuration paths for high regional innovation performance and low regional innovation performance.

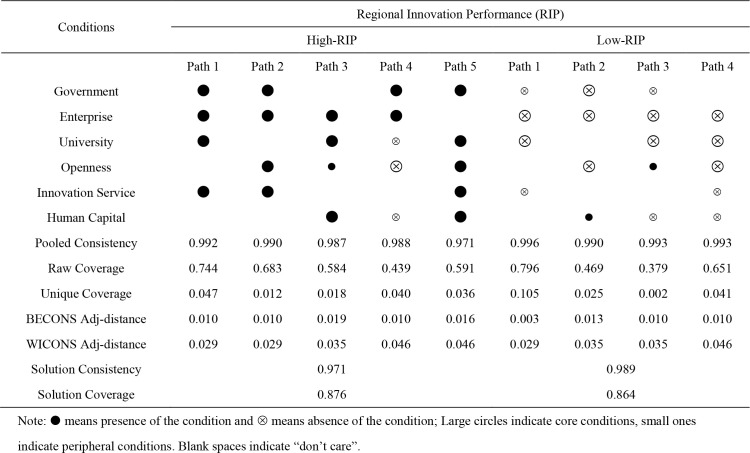

As shown in Table 5, five configurations are obtained for high-level regional innovation and four configurations for low-level regional innovation. The overall consistency values, as well as the consistency values of all configurations, are larger than 0.95, indicating that there is more than a 95% probability that high- or low-level regional innovation will be generated when the presented conditional configurations exist. Therefore, the conditional configurations derived in this study have good explanatory power for the observed results. Additionally, the solution coverage of the configurations for both high- and low-level regional innovations exceeds 0.85, indicating that the configurations cover more than 85% of the cases of high or low-level regional innovation. The configurations for high-level regional innovation, excluding Configuration 4, which has a raw coverage of 0.439, all have raw coverage values greater than 0.5, indicating that a single configuration covers more than half of the cases. In the configuration for low-level regional innovation, the raw coverage of all configurations is greater than 0.5, except for Configuration 2, which has a raw coverage of 0.469, and Configuration 3, which has a raw coverage of 0.379.

#### 4.2.4 Pooled consistency.

As shown in Table 5, Configuration 1, which leads to a high level of regional innovation, is dominated by innovation actors, with all three actors—government, enterprise, and university—forming the core conditions, with the innovation platform milieu. Configuration 1 is named “Triple-actor Collaboration with Platform Support”. This configuration embodies the “triple helix” theory, highlighting the importance of resource sharing, complementary advantages, and synergistic cooperation among government, industry, and universities in promoting regional innovation. Additionally, the establishment and enhancement of business incubators provide resources and favorable external conditions for regional innovation, particularly in business innovation, stimulating both innovation and entrepreneurship. This finding suggests that government-supported fiscal interventions should strategically prioritize fostering triple helix collaboration (industry-university-research partnerships) and expanding innovation incubator networks, thereby creating synergistic mechanisms that enhance regional innovation performance through knowledge spillovers and entrepreneurial experimentation.

Configuration 2, core conditions reflects the synergy between government and enterprises as the primary drivers, with openness and innovation platforms serving as the key milieu. Configuration 2 is named “Government-Enterprise Driven Open Innovation”. The government plays a macro-regulatory role in regional innovation, while enterprises serve as the main and most dynamic innovation agents in the market. The synergy between government and enterprises generates more innovation resources, promoting the transformation of strategic innovation plans into technology and product innovations. In the current era of strategic globalization, our findings reveal that innovation ecosystems achieve optimal performance through calibrated openness to external knowledge flows—including technology transfer, skilled labor mobility, and institutional R&D collaboration, resulting in greater market expansion. This openness dividend is further amplified by precision innovation services, collectively creating a dynamic innovation milieu that enhances both the efficiency and output quality of regional innovation actors while maintaining strategic autonomy.

Configuration 3, places enterprises, universities, and human capital at the core condition, with the open milieu as a peripheral condition. Configuration 3 is named “Enterprise-University-Human Capital Core in Open Context”. This configuration emphasizes the importance of both the origin of innovation (original research) and the development and diffusion of innovation (product innovation and market development) are essential for supporting regional innovation ecosystem. Universities cultivate talent and conduct regional innovation research, contributing primarily to original research. This university-enterprise symbiosis is further enhanced by specialized human capital that not only drives initial innovation creation but also facilitates knowledge diffusion. The open milieu condition amplifies these effects through strategic absorption of external resources, particularly in technology licensing and global talent circulation, while maintaining an optimal balance between openness and indigenous capability development that maximizes both innovation quantity and quality.

Configuration 4, the government and enterprises constitute the core conditions, the open milieu constitutes the core absent condition, and universities, human capital constitute the marginal absent conditions. Configuration 4 is named “Closed Government-Enterprise System”. This type of regional innovation is primarily driven by governments and enterprises in the absence of other conditions such as an open milieu. This configuration demonstrates that even within relatively closed economic systems, strategic public-private investment in innovation can generate high-level regional innovation performance through effective resource recombination and institutional coordination.

Configuration 5 is the government, universities, openness, innovation services, human capital as core conditions. Configuration 5 is named “Multi-Dimensional Enabling Environment for Holistic Innovation”. This configuration includes a more comprehensive set of drivers, including all three innovative milieu. This illustrates the importance of these innovative milieu and their synergies in regional innovation. The construction and improvement of a robust innovation milieu typically takes time and is characteristic of more mature regional innovation ecosystems. These systems rely on the synergistic effect of key innovation actors, including government innovation policies and resource allocation, science and technology R&D funding, and talent resources, all requiring active engagement from universities. As the foundation for knowledge creation and talent development, universities, under government guidance and support, are better positioned to carry out scientific and technological R&D, as well as provide the intellectual and talent support needed to achieve innovation policy goals.

The four low-level regional innovation configurations can be categorized into two types. The first is an enterprise–university-restricted type, which includes Configurations 1 and 3 for low-level regional innovation. Their consistency levels are 0.996 and 0.993, covering 79.6% and 37.9% of cases, respectively. The core absent conditions are enterprises, universities; this type of configuration corresponds to the core conditions of high-level regional innovation (Configuration 1). This indicates that low-level R&D inputs of enterprises and universities and their synergies can lead to low-level regional innovation.

The second type is innovation actors-openness-limitations type, which includes Configurations 2 and 4 for low-level regional innovation. These configurations have consistency levels of 0.990 and 0.993, covering 46.9% and 65.1% of the cases, respectively. The core absent conditions are the two innovation actors and an open milieu. Specifically, the core absent conditions are enterprises, universities, and openness for Configuration 4, representing all of the core and peripheral conditions for Configuration 3 for high-level regional innovation. This emphasizes that low-level regional innovation is typically associated with a closed economy. Moreover, in combination with the first type, the fundamental role of innovation actors in regional innovation can be identified.

#### 4.2.5 Between-group consistency.

**Fig 3:**
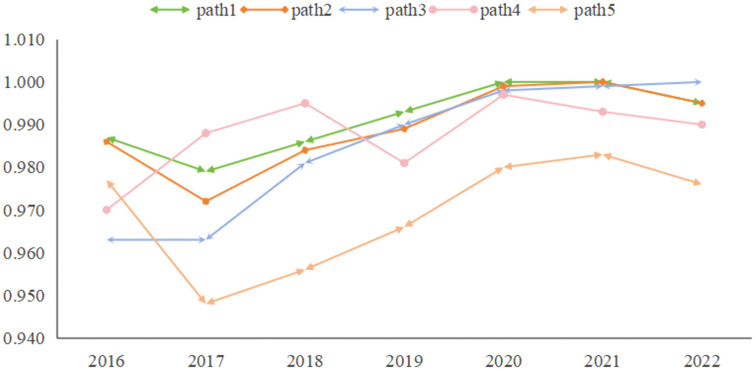
The consistency levels of the configurations vary over time.

The BECONS Adj-distance are all less than 0.2, indicating that the consistency levels of the configurations do not exhibit obvious time fluctuations; however, some change trends can still be observed. As shown in Fig 3, the consistency level of Configuration 3 exhibits an increasing trend over time and even reaches a value of one in 2022. This indicates that the explanatory strength of Configuration 3 increases over time. This configuration highlights the importance of education and human capital. Additionally, as shown in Fig 3, the consistency levels of the other configurations fluctuate slightly over time but are generally increasing. It is noteworthy that the consistency levels in 2022 are not only above the aggregated consistency level, but also above the initial level in 2016. This suggests that the results of the high-level regional innovation configurations considered in this study represent consistent developmental trends, and these factors may become increasingly important for regional innovation in China in the future.

**Fig 4:**
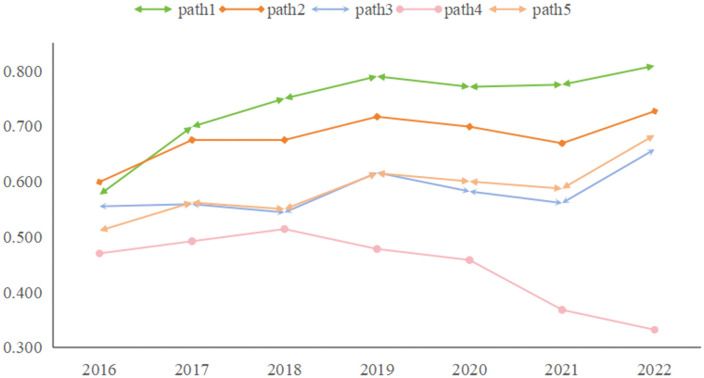
The raw coverage of the configurations varies over time.

By analyzing the temporal changes in the coverage of each configuration, we identified the trends in the number of coverage cases. As shown in Fig 4, all configurations except Configuration 4 exhibit an overall upward trend over time. The raw coverage values follow the order: Configuration 1 > 2 > 5 > 3 > 4. Configuration 1 represents regional innovation primarily driven by key innovation actors, indicating that a significant proportion of Chinese provinces and cities exhibit this type of innovation, with its coverage increasing annually. In contrast, Configuration 4, with fewer actors (only government and enterprises) and more missing conditions, explains fewer cases each year. This suggests that regional innovation is increasingly dependent on multi-factor linkages, rather than being driven by just a few factors.

Considering the historical trends and previous analysis, it can be inferred that future drivers of regional innovation will be more diverse and will exhibit multiple developments. Innovation actors have consistently played a fundamental role, and their importance has grown over time. Synergy among these actors will be crucial. For instance, increased cooperation between enterprises, academia, and the government will enhance coordination within regional innovation ecosystem and strengthen the roles of all actors. Additionally, improving innovation service platforms, fostering external openness, promoting cooperative innovation, and upgrading human capital will be vital to advancing regional innovation. With the acceleration of globalization and digitization, the degree of regional openness is likely to increase, significantly impacting regional innovation.

#### 4.2.6 Within-group consistency.

Within-group analyzes were conducted to determine whether the results of the configurations differed across regions. The primary measure is the WICONS Adj-distance, which measures whether there are significant regional differences in the level of consistency across state configurations. A consistency-adjusted distance greater than 0.2 indicates significant regional differences. After measurement, the WICONS Adj-distance of each configuration is less than 0.2 and the consistency level does not exhibit obvious differences between regions.

Although the consistency levels of the configurations across regions show little variation, examining the differences in coverage within each configuration reveals the regional distribution of cases explained by each configuration. The 30 provinces and cities in China were divided into three regions (eastern, central, and western) based on geographic location and economic development.

As shown in Table 6, the coverage values for Configurations 1–3 and 5 follow: east > west > central, indicating their stronger explanatory power for the more developed eastern region with a mature regional innovation ecosystem and efficient innovation collaboration. The western region, while less developed, benefits from government policies, making these configurations moderately applicable. The central region’s intermediate development stage results in weaker coverage. Configuration 4 shows the reverse pattern (west > central > east), as its limited conditions (only government and enterprises) better suit the western region’s underdeveloped regional innovation ecosystem. This configuration poorly explains the innovation-rich eastern region.

**Table 6:** The mean value of regional configuration coverage.

Configuration	Eastern	Central	Western
Path 1	0.809	0.645	0.748
Path 2	0.834	0.524	0.647
Path 3	0.721	0.488	0.633
Path 4	0.328	0.704	0.744
Path 5	0.732	0.502	0.626

Representative cases of Configuration 1 include Hubei, Hunan, and Henan Provinces. These provinces were historically disadvantaged by their inland locations and late economic development, lagging behind the eastern coast. However, recent efforts to prioritize innovation and optimize industrial structures have accelerated economic growth. For instance, Hubei Province, located in central China along the Yangtze River, is rich in water and mineral resources. Traditionally an agricultural region, Hubei has embraced scientific and technological innovation through policies like the 2021 Guanggu Science and Technology Innovation Corridor Development Plan. This initiative supports emerging industries such as optoelectronics, life sciences, and intelligent manufacturing. Hubei ranks among the top provinces in the number of colleges and research institutes, fostering close collaboration between academia and industry. Science-and-technology-based small and medium-sized enterprises and high-tech industries have thrived, with high-tech industries contributing over a trillion yuan in added value, accounting for more than 40% of industrial growth. The digital economy now represents 47% of GDP. Configuration 1 is characterized by active innovation actors and significant R&D investment, driven by strong demand for innovation. However, the region’s weak economic foundation and underdeveloped innovative milieu limit its progress. The regional innovation ecosystem relies heavily on inputs from actors and innovation services to sustain innovative growth.

Fujian and Guangdong Provinces exemplify Configuration 2, characterized by high openness and robust private economies. Fujian, located on the southeastern coast and designated as a pilot free-trade zone, benefits from favorable geographic positioning and strong government support for private enterprise innovation. Policies such as “Several Measures on Promoting the Innovative Development of the Private Economy in Fujian Province” have fostered scientific advancements, particularly in new energy, driving high-quality industry development. In Fujian, enterprises are the primary drivers of innovation, benefiting from strong government support and market freedom. The province’s openness facilitates the inflow of advanced knowledge and technology, enabling rapid transformation of innovations into production benefits. This highly marketized regional innovation ecosystem is propelled by enterprise innovation, yet cultural and educational contributions, including universities and human capital, remain less prominent. To enhance innovation capacity, it is recommended that Fujian strengthen collaboration among industries, universities, and research institutes, while improving human capital development. These efforts will provide a stronger talent base and support sustainable, long-term innovation development.

Zhejiang Province and Tianjin City exemplify Configuration 3, marked by R&D, human capital, and strong collaboration among innovation actors. Zhejiang, located on China’s southeast coast, boasts a favorable ecological environment and deep cultural heritage. It hosts numerous high-level universities, key laboratories, and research centers, which drive innovation through close cooperation with enterprises. Zhejiang has developed advanced manufacturing and modern service industries, fostering innovative enterprises and competitive industrial clusters. Traditional industries, such as silk and tea, have been upgraded, while the rapidly expanding digital economy propels industrial innovation. With its openness, Zhejiang establishes platforms like free-trade zones to attract investments and cultivate talent. Through policy support and infrastructure, it has built a diversified and high-quality human capital base. The primary drivers of innovation in Zhejiang are its education system, human capital, and collaboration among industries, universities, and research institutes. This regional innovation ecosystem leverages its strong R&D capabilities to ensure long-term innovation and development. However, further strengthening synergies among innovation actors and enhancing service platforms is needed to better align innovation processes with strategic goals and outcomes.

Representative cases of Configuration 4 include Guizhou and Yunnan provinces. Guizhou Province is located in the inland hinterland of Southwest China and has predominantly mountainous terrain. It has a low degree of openness to the outside world and a weak economic foundation. In recent years, the Guizhou provincial government has actively promoted the construction of an innovation system with a focus on taking advantage of the unique advantages of the big data industry. It has introduced policies, including the “Implementation Plan for Promoting the Deep Integration of Big Data and Industry, and Developing the Industrial Internet in Guizhou Province” to develop the big data industry (source from Guizhou Provincial People’s Government Website). The rapid development of the big data industry has also enhanced regional innovation capacity and driven the development of other industries. However, the number and quality of colleges and universities in Guizhou are relatively limited, and there is an inadequate talent structure and shortage of talent in high-tech fields. Additionally, Guizhou’s innovative milieu needs to be optimized and upgraded. Overall, Guizhou Province possesses relatively few innovation advantages and resources, and remains a relatively closed innovation system, primarily driven by the government and enterprises.

The representative cases of Configuration 5 include the Beijing Municipality. Beijing is the capital of China, possessing a high level of economic development, rich innovation resources, and a favorable innovative milieu. The drivers of regional innovation included in Configuration 5 are the most comprehensive among all configurations, reflecting that Beijing has a relatively complete regional innovation ecosystem. Beijing’s regional innovation resources are primarily focused on the sources of innovation, namely knowledge and originality. This characteristic is reflected in the number of scientific research institutions, scientific research talent, scientific research funds, and sectoral distribution of scientific and technological outputs in Beijing. The innovative approach is based on originality and emphasizes foundational research and advancements in cutting-edge technology. Innovation activities have a significant knowledge spillover and radiation effect, which can drive the development of science and technology innovation in neighboring regions and the country as a whole. Beijing also emphasizes comprehensive and coordinated development, including the in-depth integration of scientific and technological innovation with industrial upgrades, urban development, and social governance.

## 5. Discussion

First, our research reveals that strong government innovation actors are necessary for high-level regional innovation, while weak enterprise innovation actors are necessary for non-high regional innovation performance. This finding advances understanding of the government-market relationship in regional innovation systems. This finding aligns with the statist model of the Triple Helix framework by Etzkowitz and Leydesdorff [17], where governments control academia and industry, lead project development, and offer resource support for new initiatives. Moreover, it empirically validates Cooke’s claim that regulation is crucial for regional innovation [10].

Second, configuration 4 (government and enterprises with a lack of openness) supplements the open innovation theory. Existing literature emphasizes openness and external knowledge acquisition for regional competitiveness [70,71]. But this configuration shows that in some regional contexts, a relatively closed government-enterprise collaborative system can also achieve high innovation performance. It doesn’t deny the value of openness but refines its applicability: strong internal resource integration and institutional support can partly substitute external knowledge reliance. Further analysis shows this path explains western Chinese regions in early technological catch-up stages, which adopt a mission-driven.

Third, this study enhances the understanding of the Triple Helix theory through a comparative analysis of various configurations. Configuration 1 confirms the university-industry-government tripartite collaboration as an optimal model [17]. In contrast, Configuration 2 (government-enterprise collaboration) and Configuration 3 (enterprise-university-human capital collaboration) show that regional innovation doesn’t always require perfect tripartite coordination. Instead, it can be driven by powerful combinations between two actors. This finding shifts the analytical focus from “the presence of three actors” to “how actors achieve functional complementary and effective interaction,” supporting Cai & Etzkowitz’s claim [71] that the Triple Helix shows structural variation.

Fourth, the measurement results show that innovation performance in all regions has improved over time. The between-group consistency reveals that regional innovation increasingly depends on complex interactions among multiple actors and environments. Within-group consistency indicates that configurations 1, 2, and 3 have stronger explanatory power in eastern regions than in central and western regions, while configuration 4 has greater explanatory power in western regions. Configuration 4 differs from others with its closed system and deficient innovation environment. This implies that in developed eastern regions, innovation is driven by multiple innovation actors and environments, with high external knowledge dependence due to openness. In contrast, innovation in underdeveloped western regions mainly relies on internal drivers like government and enterprise innovation investments. Balland [72] pointed out that previous research had not sufficiently explored the region-specific knowledge structures and evolutionary stages, and our findings contribute to filling this research gap. These findings enhance the understanding of innovation driven mechanisms at different stages and offer references for targeted innovation policies.

## 6. Conclusion, implication and generalization of the research

### 6.1 Conclusion

Drawing on CAS theory, this study investigates the dynamic evolution and configurational pathways of regional innovation ecosystems. First, we quantitatively assess regional innovation performance across Chinese provinces, with comparative analysis of eastern, central and western regions. Next we apply a novel dynamic fsQCA approach to identify evolutionary configuration pathways of regional innovation, including identifying the necessary and sufficient conditions by constructing conditional variables from key innovation actors and their development. Furthermore, we examined the temporal trends in these configurations and analyzed regional differences, as well as representative cases.

The main findings of this research are the following five aspects: First, based on the average values from 2016 to 2022, the top five provinces with the highest regional innovation performance were Guangdong, Jiangsu, Zhejiang, Shandong, and Beijing, while the bottom five were Gansu, Xinjiang, Ningxia, Hainan, and Qinghai. Regional innovation performance in all provinces improved over time. Second, the eastern region exhibited the highest regional innovation performance, followed by the central region, and then the western region. Third, the subsequent analysis of regional innovation drivers revealed that a strong government innovation actor is a necessary condition for high-level regional innovation, while a weak enterprise innovation actor is a necessary condition for low-level regional innovation. Fourth, we identified five configurations driving high-level regional innovation and four driving low-level regional innovation. The configurations driving high-level regional innovation include: Triple-actor Collaboration with Platform Support; Government-Enterprise Driven Open Innovation; Enterprise-University-Human Capital Core in Open Context; Closed Government-Enterprise System; and Multi-Dimensional Enabling Environment for Holistic Innovation. Finally, between-group results suggest that the high-level regional innovation configurations may become increasingly important for regional innovation in the future, and regional innovation is increasingly dependent on multi-factor linkages, rather than being driven by just a few factors. Through within-group analysis, we observed that configurations 1, 2, 3, and 5 followed the order of east> west> central, while configuration 4 followed the order of west> central> east.

### 6.2 Practice implications

Based on our findings, we propose the following policy recommendations: First, R&D investment and collaboration among innovation actors are foundational for all types of regional innovation systems. Second, in regions experiencing rapid economic growth and benefiting from policy dividends, strengthening investments in and coordination among innovation actors, while prioritizing innovation platform development to facilitate greater innovation resource flows, can capitalize on policy advantages and promote the efficiency of regional innovation ecosystems. Third, in regions with high openness and market activity, it is crucial to maintain institutional flexibility and balanced regulation while enhancing government-enterprise collaboration to sustain robust innovation momentum. By leveraging openness to attract external knowledge, technologies, and capital investments, while concurrently strengthening internal innovation platform development, regional innovation can be effectively advanced. Fourth, in regions with abundant educational and cultural resources, promoting closer enterprise and university collaboration, increasing R&D investment, advancing original innovation and technology commercialization, and continuously developing human capital can enhance the long term innovative capacity of the innovation ecosystem. Fifth, mature regional innovation systems will be crucial for sustaining regional innovation growth, particularly by further developing a robust innovation environment and culture, including innovation platforms, openness, and human capital.

### 6.3 Generalization of the research

Although the theoretical framework and empirical findings of this study are based on the Chinese context, the employed CAS theory framework and dynamic QCA possess universal applicability, contributing value to relevant research in other regions. Consequently, their relevance and application can be extended beyond China, especially to economies in the process of rapid industrialization, technological catch-up, or economic transformation.

First, drawing on the CAS theory, this study reveals the laws governing the synergistic influence of innovation actors and the environment on regional innovation. In both mature innovation economies and emerging innovation regions, innovation ecosystems are intricate systems composed of actors such as enterprises, universities, and governments, which adapt, learn, and interact under environmental factors. Therefore, the actor and environment co-evolutionary analysis framework provides a transferable paradigm for other regions. Second, the dynamic QCA method can discern the impact of different configurations of multiple conditions on outcomes. It is appropriate for analyzing the complex effects of diverse regional conditions on innovation performance, which is precisely what regions with different backgrounds need. Finally, the findings of this study can be translated into adaptable policy design principles for decision makers. For example, the positioning of government roles, the diversity of innovation models, and the spatial-temporal heterogeneity of innovation driven mechanisms hold practical reference value.

### 6.4 Limitations and future research

This study has limitations. First, regional innovation is influenced by diverse and variable factors, including economy, society, culture, and policy. While key subjects and milieu factors are considered, not all variables or interactions are captured. Second, data constraints restricted the study’s temporal scope, with some variables excluded due to data quality or availability. Future research should incorporate sociocultural and policy dimensions to explore factor interactions more comprehensively. Expanding the dataset’s time-span, coverage, and granularity could further elucidate the dynamic processes of regional innovation.

## Supporting information

S1 File Data. (XLSX)

## References

[1] OECD. The Innovation Imperative: Contributing to Productivity, Growth and Well-Being. OECD Publishing, Paris; 2015. 10.1787/9789264239814-en

[2] Dutta S, Lanvin B, Rivera León L, Wunsch-Vincent S. Global Innovation Index 2023: Innovation in the face of uncertainty. World Intellectual Property Organization. 2023. doi: 10.34667/TIND.48220

[3] McCann P, Ortega-Argiles R. Modern regional innovation policy. Cambridge Journal of Regions, Economy and Society. 2013;6(2):187–216. doi: 10.1093/cjres/rst007

[4] Cooke P, Gomez Uranga M, Etxebarria G. Regional innovation systems: Institutional and organisational dimensions. Research Policy. 1997;26(4–5):475–91. doi: 10.1016/s0048-7333(97)00025-5

[5] Chung S. Building a national innovation system through regional innovation systems. Technovation. 2002;22(8):485–91. doi: 10.1016/s0166-4972(01)00035-9

[6] Asheim BT, Smith HL, Oughton C. Regional Innovation Systems: Theory, Empirics and Policy. Regional Studies. 2011;45(7):875–91. doi: 10.1080/00343404.2011.596701

[7] Chen W, Song H. National innovation system: Measurement of overall effectiveness and analysis of influencing factors. Technology in Society. 2024;77:102514. doi: 10.1016/j.techsoc.2024.102514

[8] Mu Q, Lee K. Knowledge diffusion, market segmentation and technological catch-up: The case of the telecommunication industry in China. Research Policy. 2005;34(6):759–83. doi: 10.1016/j.respol.2005.02.007

[9] Du Z-Y, Wang Q. Digital infrastructure and innovation: Digital divide or digital dividend?. Journal of Innovation & Knowledge. 2024;9(3):100542. doi: 10.1016/j.jik.2024.100542

[10] Cooke P. Regional innovation systems: Competitive regulation in the new Europe. Geoforum. 1992;23(3):365–82. doi: 10.1016/0016-7185(92)90048-9

[11] Granstrand O, Holgersson M. Innovation ecosystems: A conceptual review and a new definition. Technovation. 2020;90–91:102098. doi: 10.1016/j.technovation.2019.102098

[12] Fritsch M, Slavtchev V. Universities and Innovation in Space. Industry and Innovation. 2007;14(2):201–18. doi: 10.1080/13662710701253466

[13] Hollanders H, Es-Sadki N. Regional Innovation Scoreboard 2021. European Commission, Directorate-General for Internal Market, Industry, Entrepreneurship and SMEs. Available from: https://ec.europa.eu/docsroom/documents/46031

[14] Boschma R. Towards an Evolutionary Perspective on Regional Resilience. Regional Studies. 2014;49(5):733–51. doi: 10.1080/00343404.2014.959481

[15] Freeman C. Networks of innovators: A synthesis of research issues. Research Policy. 1991;20(5):499–514. doi: 10.1016/0048-7333(91)90072-x

[16] Emergence of a Triple Helix of university—industry—government relations. Science and Public Policy. 1996. doi: 10.1093/spp/23.5.279

[17] Etzkowitz H, Leydesdorff L. The dynamics of innovation: from National Systems and “Mode 2” to a Triple Helix of university–industry–government relations. Research Policy. 2000;29(2):109–23. doi: 10.1016/s0048-7333(99)00055-4

[18] Binz C, Truffer B, Coenen L. Why space matters in technological innovation systems—Mapping global knowledge dynamics of membrane bioreactor technology. Research Policy. 2014;43(1):138–55. doi: 10.1016/j.respol.2013.07.002

[19] Grillitsch M, Trippl M. Combining Knowledge from Different Sources, Channels and Geographical Scales. European Planning Studies. 2013;22(11):2305–25. doi: 10.1080/09654313.2013.835793

[20] Coenen L, Benneworth P, Truffer B. Toward a spatial perspective on sustainability transitions. Research Policy. 2012;41(6):968–79. doi: 10.1016/j.respol.2012.02.014

[21] Binz C, Truffer B. Global Innovation Systems—A conceptual framework for innovation dynamics in transnational contexts. Research Policy. 2017;46(7):1284–98. doi: 10.1016/j.respol.2017.05.012

[22] Binz C, Truffer B, Coenen L. Path Creation as a Process of Resource Alignment and Anchoring: Industry Formation for On-Site Water Recycling in Beijing. Economic Geography. 2015;92(2):172–200. doi: 10.1080/00130095.2015.1103177

[23] Zhang J, Li J, Yan Y, Xie Z. Concentration in cross‐border research collaborations and MNCs’ knowledge creation in a host country. Strategic Management Journal. 2025. doi: 10.1002/smj.70025

[24] Freeman C. Technology and Economic Performance: Lessons from Japan. London: Pinter Publishers; 1987.

[25] Patel P, Pavitt K. National Innovation Systems: Why They Are Important, And How They Might Be Measured And Compared. Economics of Innovation and New Technology. 1994;3(1):77–95. doi: 10.1080/10438599400000004

[26] Nelson RR, editor. National innovation systems: a comparative analysis. New York: Oxford University Press; 1993.

[27] Hu Y, Liu D. Government as a non-financial participant in innovation: How standardization led by government promotes regional innovation performance in China. Technovation. 2022;114:102524. doi: 10.1016/j.technovation.2022.102524

[28] Anokhin S, Wincent J, Parida V, Chistyakova N, Oghazi P. Industrial clusters, flagship enterprises and regional innovation. Entrepreneurship & Regional Development. 2018;31(1–2):104–18. doi: 10.1080/08985626.2018.1537150

[29] Thomas E, Faccin K, Asheim BT. Universities as orchestrators of the development of regional innovation ecosystems in emerging economies. Growth and Change. 2020;52(2):770–89. doi: 10.1111/grow.12442

[30] Krieger B. Heterogeneous university funding programs and regional firm innovation. Research Policy. 2024;53(5):104995. doi: 10.1016/j.respol.2024.104995

[31] Lehmann EE, Stockinger SAE. Entrepreneurship in Higher Education: The impact of competition‐based policy programmes exemplified by the German Excellence Initiative. Higher Education Quarterly. 2018;73(1):70–84. doi: 10.1111/hequ.12188

[32] Crevoisier O. The Innovative Milieus Approach: Toward a Territorialized Understanding of the Economy?. Economic Geography. 2004;80(4):367–79. doi: 10.1111/j.1944-8287.2004.tb00243.x

[33] Wang J, Yang N, Guo M. How social capital influences innovation outputs: an empirical study of the smartphone field. Innovation. 2020;23(4):449–69. doi: 10.1080/14479338.2020.1810580

[34] Pfotenhauer SM, Wentland A, Ruge L. Understanding regional innovation cultures: Narratives, directionality, and conservative innovation in Bavaria. Research Policy. 2023;52(3):104704. doi: 10.1016/j.respol.2022.104704

[35] Dai Y, Tong X, Jia X. Executives’ Legal Expertise and Corporate Innovation. Corporate Governance. 2024;32(6):954–83. doi: 10.1111/corg.12578

[36] Hasan I, (Stan) Hoi C-K, Wu Q, Zhang H. Is social capital associated with corporate innovation? Evidence from publicly listed firms in the U.S. Journal of Corporate Finance. 2020;62:101623. doi: 10.1016/j.jcorpfin.2020.101623

[37] Zhang L, Huang S. Social capital and regional innovation efficiency: The moderating effect of governance quality. Structural Change and Economic Dynamics. 2022;62:343–59. doi: 10.1016/j.strueco.2022.05.013

[38] Holland JH. Hidden order: how adaptation builds complexity. Basic Books Press; 1996.

[39] Mitchell M. Complexity: A Guided Tour. Oxford University Press. 2009.

[40] Arthur W. Complexity and the economy. Science. 1999;284(5411):107–9. doi: 10.1126/science.284.5411.107 10103172

[41] Levin SA. Ecosystems and the Biosphere as Complex Adaptive Systems. Ecosystems. 1998;1(5):431–6. doi: 10.1007/s100219900037

[42] Martin R, Sunley P. Path dependence and regional economic evolution. Journal of Economic Geography. 2006;6(4):395–437. doi: 10.1093/jeg/lbl012

[43] Castro RG, Ariño MA. A General Approach to Panel Data Set-Theoretic Research. JAMSIS. 2016;2:63–76. doi: 10.6000/2371-1647.2016.02.06

[44] Finn V. A qualitative assessment of QCA: method stretching in large-N studies and temporality. Qual Quant. 2022;56(5):3815–30. doi: 10.1007/s11135-021-01278-5

[45] Guedes MJ, da Conceição Gonçalves V, Soares N, Valente M. UK evidence for the determinants of R&D intensity from a panel fsQCA. Journal of Business Research. 2016;69(11):5431–6. doi: 10.1016/j.jbusres.2016.04.150

[46] Kent R, Olsen W. Using fsQCA a Brief Guide and Workshop for Fuzzy-Set Qualitative Comparative Analysis. Department of Marketing University of Stirling; 2008. Available from: http://hummedia.manchester.ac.uk/institutes/cmist/archive-publications/working-papers/2008/2008-10-teaching-paper-fsqca.pdf

[47] Schneider CQ, Wagemann C. Standards of Good Practice in Qualitative Comparative Analysis (QCA) and Fuzzy-Sets. Comp Sociol. 2010;9(3):397–418. doi: 10.1163/156913210x12493538729793

[48] Huang Z. Research on Innovation Capability of Regional Innovation System Based on Fuzzy-Set Qualitative Comparative Analysis: Evidence from China. Systems. 2022;10(6):220. doi: 10.3390/systems10060220

[49] Ragin CC. Redesigning social inquiry: Fuzzy sets and beyond. University of Chicago Press. 2009.

[50] Cai Z, Ma D, Zhou R, Zhang Z. Unraveling the impact of patent transfers on regional innovation: Empirical insights through the lens of entity relationships. Technological Forecasting and Social Change. 2024;208:123666. doi: 10.1016/j.techfore.2024.123666

[51] Ejermo O. Regional Innovation Measured by Patent Data—Does Quality Matter?. Industry and Innovation. 2009;16(2):141–65. doi: 10.1080/13662710902764246

[52] Quatraro F. Diffusion of Regional Innovation Capabilities: Evidence from Italian Patent Data. Regional Studies. 2008;43(10):1333–48. doi: 10.1080/00343400802195162

[53] Fu W, Diez JR. Knowledge spillover and technological upgrading: The case of Guangdong province, China. Asian Journal of Technology Innovation. 2010;18(2):187–217. doi: 10.1080/19761597.2010.9668698

[54] Li T, Fu W. Spatial processes of regional innovation in Guangdong Province, China: empirical evidence using a spatial panel data model. Asian Journal of Technology Innovation. 2015;23(3):304–20. doi: 10.1080/19761597.2015.1120499

[55] Rapini MS, Chiarini T, Stein A de Q. Universities in inclusive regional innovation systems: Academic engagement and uneven knowledge use in Brazil. Journal of Regional Science. 2023;64(1):108–35. doi: 10.1111/jors.12667

[56] Zhang M, Li B, Yin S. Configurational paths to regional innovation performance: the interplay of innovation elements based on a fuzzy-set qualitative comparative analysis approach. Technology Analysis & Strategic Management. 2020;32(12):1422–35. doi: 10.1080/09537325.2020.1773423

[57] Li X. China’s regional innovation capacity in transition: An empirical approach. Research Policy. 2009;38(2):338–57. doi: 10.1016/j.respol.2008.12.002

[58] Fernández-Macho J, González P, Virto J. Assessing anthropogenic vulnerability of coastal regions: DEA-based index and rankings for the European Atlantic Area. Marine Policy. 2020;119:104030. doi: 10.1016/j.marpol.2020.104030

[59] Jiao H, Zhou J, Gao T, Liu X. The more interactions the better? The moderating effect of the interaction between local producers and users of knowledge on the relationship between R&D investment and regional innovation systems. Technological Forecasting and Social Change. 2016;110:13–20. doi: 10.1016/j.techfore.2016.03.025

[60] Teirlinck P, Spithoven A. The Spatial Organization of Innovation: Open Innovation, External Knowledge Relations and Urban Structure. Regional Studies. 2008;42(5):689–704. doi: 10.1080/00343400701543694

[61] Ning L, Wang F, Li J. Urban innovation, regional externalities of foreign direct investment and industrial agglomeration: Evidence from Chinese cities. Research Policy. 2016;45(4):830–43. doi: 10.1016/j.respol.2016.01.014

[62] Xiao L, North D. The graduation performance of technology business incubators in China’s three tier cities: the role of incubator funding, technical support, and entrepreneurial mentoring. J Technol Transf. 2016;42(3):615–34. doi: 10.1007/s10961-016-9493-4

[63] Wang Z, He Q, Xia S, Sarpong D, Xiong A, Maas G. Capacities of business incubator and regional innovation performance. Technological Forecasting and Social Change. 2020;158:120125. doi: 10.1016/j.techfore.2020.120125

[64] Lucas RE Jr. On the mechanics of economic development. Journal of Monetary Economics. 1988;22(1):3–42. doi: 10.1016/0304-3932(88)90168-7

[65] Romer PM. Endogenous Technological Change. Journal of Political Economy. 1990;98(5, Part 2):S71–102. doi: 10.1086/261725

[66] Barro RJ, Lee JW. International measures of schooling years and schooling quality. Am Econ Rev. 1996;86:218–23.

[67] Huang L, Liu X, Xu L. Regional Innovation and Spillover Effects of Foreign Direct Investment in China: A Threshold Approach. Regional Studies. 2012;46(5):583–96. doi: 10.1080/00343404.2010.520694

[68] Andrews R, Beynon MJ, McDermott AM. Organizational Capability in the Public Sector: A Configurational Approach. Journal of Public Administration Research and Theory. 2015;26(2):239–58. doi: 10.1093/jopart/muv005

[69] Fiss PC. Building Better Causal Theories: A Fuzzy Set Approach to Typologies in Organization Research. AMJ. 2011;54(2):393–420. doi: 10.5465/amj.2011.60263120

[70] Schmidt S, Müller FC, Ibert O, Brinks V. Open Region: Creating and exploiting opportunities for innovation at the regional scale. European Urban and Regional Studies. 2017;25(2):187–205. doi: 10.1177/0969776417705942

[71] Cai Y, Etzkowitz H. Theorizing the Triple Helix model: Past, present, and future. Triple Helix. 2020;7(2–3):189–226. doi: 10.1163/21971927-bja10003

[72] Balland P-A, Boschma R, Crespo J, Rigby DL. Smart specialization policy in the European Union: relatedness, knowledge complexity and regional diversification. Regional Studies. 2018;53(9):1252–68. doi: 10.1080/00343404.2018.1437900

